# Delay adaptation does not transfer between discrete button press actions and continuous control

**DOI:** 10.1177/20416695251352067

**Published:** 2025-07-02

**Authors:** Loes CJ van Dam, Svenja Kernig, Karina Lazarova, Melisa Ünal, Nicole Gappa, Benjamin Straube, Thomas SA Wallis

**Affiliations:** 1 Technical University of Darmstadt (TU Darmstadt), Department of Human Sciences, Institute for Psychology/Centre for Cognitive Science, Darmstadt, Germany; 2 Center for Mind, Brain and Behavior, University of Marburg, Justus Liebig University Giessen, Technical University of Darmstadt, Marburg, Germany; 3 Department of Psychology, 2591 University of Essex, Colchester, UK; 4 Department of Psychiatry and Psychotherapy, University of Marburg, Marburg, Germany

**Keywords:** visuomotor control, target tracking, delay adaptation, adaptation transfer

## Abstract

When interacting with technology, humans often deal with delays between an action and the desired action outcome. Through delay adaptation these delays will become less detrimental to visuomotor performance over time. Delay adaptation has been shown for a variety of tasks and control modes, from simple button presses causing a beep or flash to continuous target-tracking tasks. Here we investigated whether the delay adaptation is specific for the control mode used, when the task itself remained unaltered. To this end, participants performed a target tracking task in which they controlled a cursor item either by moving a stylus on a graphics tablet or by pressing the arrow keys on a keyboard. We found that delay adaptation occurred for both these types of control modes, but observed no transfer to the other control mode. This indicates that delay adaptation is specific to the control mode used during adaptation.

## How to cite this article

Loes C. J. van Dam, Svenja Kernig, Karina Lazarova, Melisa Ünal, Nicole Gappa, Benjamin Straube, & Thomas S. A. Wallis (2025). Delay adaptation does not transfer between discrete button press actions and continuous control. *i-Perception*, 16(4), 1–9. https://doi.org/10.1177/20416695251352067

## Introduction

For everyday actions, such as grasping a cup of coffee, it is important to keep our sensorimotor system well calibrated in both space and time. When there is a sudden disturbance, our system will need to adapt to it to compensate. With the increased use of technology, we are more and more frequently exposed to disturbances of a temporal kind, as any system needs time to process user input to provide corresponding output to the user. The user will generally experience this as a sensorimotor delay between initiating an action and sensing the expected sensory feedback of that action. The consequences of such delays vary between being annoying, e.g. a computer being slow to register a key-press, to outright dangerous such as in medical tele-operations since delays can cause the operator to overshoot the movement target (Botzer & Karniel, 2013; Rohde et al., 2014). Luckily, humans are able to effectively adapt to such delays in a variety of circumstances (e.g. de la Malla et al., 2014; Rohde et al., 2014; Stetson et al., 2006).

What is not well known, however, is to what extent delay adaptation generalises to different contexts than the one in which the adaptation occurred. For discrete actions like button presses, it was found that delay adaptation can transfer between visual, auditory and tactile feedback modalities (Arikan et al., 2021; Heron et al., 2009; Sugano et al., 2010), suggesting that the adaptation to some degree resides within the motor control component. However, when varying the motor control component, in more continuous goal-oriented motor tasks, the literature on the extent to which delay adaptation generalises is more mixed. One of the most prominent works in this direction is a study by de la Malla et al. (2014) that investigated delay adaptation in computerised target-interception tasks. They observed that participants were able to compensate for the delay when intercepting moving targets and then tested whether the adaptation effect transferred to similar tasks. Their results showed partial transfer in conditions that tested different spatial extents of the same task, i.e. when varying the initial distance to the target. However, in most cases transfer seemed largely absent, for instance, when having to move through a moving gap instead of intercepting a moving target, reversing the movement direction, or when synchronising the unseen hand to a sinusoidally moving target. Interestingly, Rohde et al. (2014) did find transfer to this sinusoidal movement synchronisation task after delay adaptation in a continuous target-tracking task. In that study participants tracked a moving target with a delayed cursor, whilst the target movements were rendered predictable by showing its future trajectory ahead of time. Though transfer was observed for the synchronisation task in this case, there was no transfer to a condition where the target movement was unpredictable.

Why does delay adaptation transfer between some conditions but not to others? We here propose that the movement control conditions play a crucial role. In the study by Rohde et al. (2014) the predictable target allowed for control based on predicting future states. The same can be said for the synchronisation task for which the movement of the target becomes predictable after one cycle, and can therefore rely on the same control mode. In contrast, the different tasks in de la Malla et al. (2014) varied along more dimensions, for instance, in terms of the phase within a movement (early or late) that was relevant for the interception and through this the extent to which sensory feedback can be taken into account, and direction of the movement (forward or backward). The lack of transfer between such conditions is analogous to similar findings when adapting to spatial offsets, where specificity was observed for movement phase, movement type or trajectory, and hand used (e.g. Kaernbach et al., 2002; Martin et al., 1996; Prablanc et al., 1975; van Dam & Ernst, 2015). It is therefore feasible that delay adaptation is specific to the control mode used during adaptation.

To test this hypothesis, we present a target-tracking experiment in which participants adapted to a visuomotor delay using one of two different control modes for moving a cursor item. The cursor could be moved either continuously by moving a stylus on a graphics tablet (Stylus Mode) or by using the arrow keys on a regular keyboard which moved the cursor in discrete steps (Buttons Mode). The visual display in the two conditions was otherwise identical. In this task, a change in tracking lag (temporal error) after adaptation indicates a temporal adjustment of behaviour (Rohde et al., 2014). Consistent with the idea that delay adaptation is control-mode specific, we found such adaptation aftereffects for both control modes when adapted directly, but a lack of transfer between these modes.

## Methods

### Participants

Eighteen participants volunteered for the experiment (age range = [19, 58] years, mean age 25, 13 female, all right-handed, all had normal or corrected-to-normal vision). Five participants (five authors) were involved in the development and execution of the experiment. All other participants were naive as to the purpose of the experiment. Informed consent was obtained from participants before starting the task.

### Apparatus

**Figure 1.**
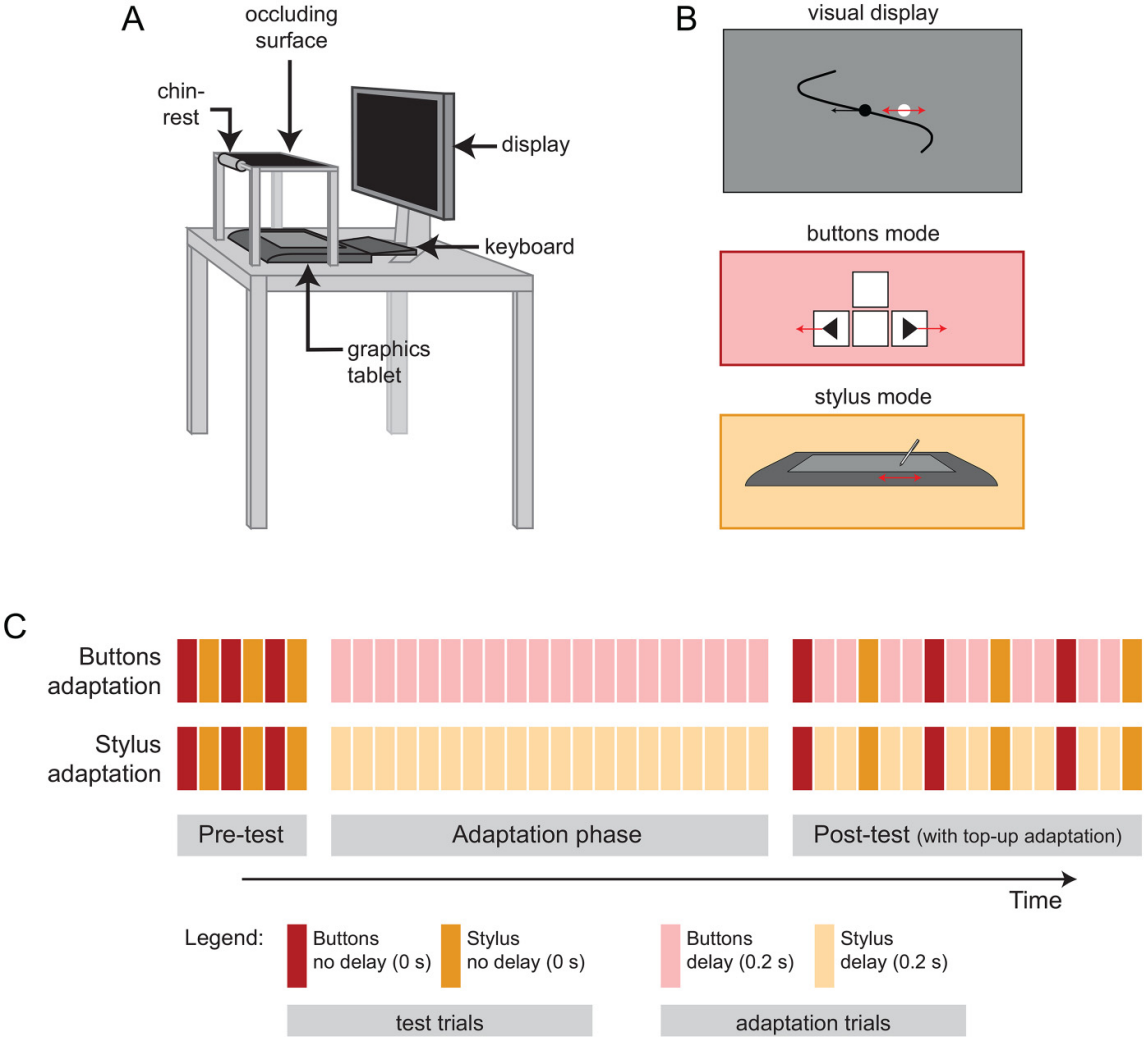
Setup and procedure. (A) Setup. (B) Participants tracked the target (black dot) as closely as possible with the cursor (white dot). The cursor was controlled using either the left and right buttons on a regular keyboard, or a graphics tablet. (C) Procedure. A pre-test including both Buttons and Stylus test trails was followed by an adaptation phase (using either Buttons or Stylus mode in two separate blocks) in which a 200 ms feedback delay was added to the cursor. The post-test alternated between Buttons and Stylus trials with 0 ms delay, interleaved with top-up adaptation trials to avoid de-adaptation. All participants completed both the Stylus and Buttons adaptation blocks. The order of the two adaptation blocks was counterbalanced across participants to counteract order effects.

The experiment was run on a Windows 10 Pro desktop computer (with CPU: AMD Ryzen 7 3700X, GPU: NVIDIA GeForce RTX 3060 graphics card and 16GB DDR4 RAM). The experiment was implemented using the Psychopy (Peirce, 2009) library in Python 3.9. The stimuli were presented on a gaming monitor (model AOC Q27G2U, 27 inch diagonal, 2560 by 1440 pixels, display area 596.736 by 335.664 mm, 144 Hz refresh rate). Cursor-control happened by moving a stylus on a graphics tablet (Wacom Intuos Pro Large, system delay 37 ± 9 ms as measured using the method described in Di Luca (2010)) or by pressing the left and right arrow keys on a standard keyboard (system delay 16 ± 7 ms). The active area of the graphics tablet (311 by 216 mm) was mapped to the screen such that the corners of the active area corresponded to the corners of the screen. Direct visibility of these input devices and the hand was prevented by a board placed over them, which doubled as a chin-rest (Figure 1). The viewing distance was approximately 50 cm.

### Stimuli

Participants performed a manual target-tracking task adapted from Rohde et al. (2014). They were presented with a moving black circular target (radius 4.7 mm) and a white circular cursor (radius 2.3 mm) presented on an intermediate grey background. The movements of target and cursor were restricted to the horizontal direction. Vertically both target and cursor were presented in the middle of the screen. The movement of the target consisted of a sum of five sine waves with frequencies 0.09, 0.165, 0.195, 0.375, and 0.495 Hz, each initialised with a random starting phase. This lead to a relatively smooth path with limited predictability. Each sinus was given an amplitude of 9.3 mm, thus the maximum horizontal position of the target was 46.6 mm left or right from the centre of the screen. To add predictability to the target movement, the target’s trajectory (width 2.3 mm) was provided within a temporal window of 1 second into the future to 1 second into the past. Spatially, this path extended 46.6 mm above and below the centre of the screen (see Figure 1).

The participants’ task was to follow the target as accurately as possible with the cursor. However, the mode for controlling the cursor differed across trials. For the Stylus Mode participants controlled the cursor using a stylus on a graphics tablet. For the Buttons Mode participants used the left and right arrow keys on the keyboard to induce jumps of the cursor to the left or right correspondingly. One button press moved the cursor by a single step of 7.0 mm. This step-size was chosen during a pilot phase such that participants would be able to follow the target by repeatedly pressing the buttons as well as visually distinguish the corresponding cursor jumps. Importantly, participants were instructed to use the same hand for both control modes. Participants were informed which mode to use for the next trial through written instructions on the screen before the trial started. For example Button and Stylus trials see Supplemental Videos.

### Procedure

Participants performed two blocks of trials that each consisted of a pre-test, adaptation phase, and post-test (see Figure 1C).

In the pretest participants alternately performed 3 Buttons trials and 3 Stylus trials, all without added feedback delay (6 pre-test trials in total). In both blocks the pre-test started with a Buttons trial. Pre-test trials lasted 10 seconds each.

During the primary adaptation phase (20 trials), participants performed the same task while a delay of 200 ms was added to the visual cursor feedback. Adaptation trials lasted 20 seconds each and in the separate blocks were either all Buttons trials (Buttons Adaptation) or all Stylus trials (Stylus Adaptation).

The post-test included 3 Buttons test trials and 3 Stylus test trials in alternate fashion (10 seconds each, starting with a Buttons trial) and without feedback delay. To prevent de-adaptation during the post-test, consecutive test-trials were interleaved with two top-up adaptation trials (20 seconds, 200 ms delay) using the corresponding mode for that block.

The two experimental blocks differed only in terms of the control mode used during adaptation trials. All participants completed both the Stylus Adaptation block and the Buttons Adaptation block. The order of the two blocks was counterbalanced across participants. Before starting the experiment, participants practised both Stylus and Buttons control modes, until both participant and experimenter were satisfied that the task was understood.

Figure S1 in the Supplemental Materials shows example participant trajectories for key trials in the procedure.

### Analysis

Target and cursor positions were recorded at the refresh rate of the display (144 Hz). Trials for which there were fewer than 5 unique samples for the cursor were removed from further analysis (4 out of 1512 trials across all participants). This seems to have happened when participants realised too late that the control mode for a given trial was different from the one they were expecting, despite the instruction before the trial.

For each remaining trial, we disregarded the first 2 seconds as participants were still aligning to the target. The remainder of the samples were analysed in terms of temporal error between the target and cursor positions. We obtained the temporal error using the cross-correlation technique: the trajectory of the cursor is shifted backwards and forwards in time (one sample at a time up to 1 second in either direction) and for each shift the correlation between target and cursor positions was calculated. The time-shift that leads to the maximum correlation between target and cursor identifies the temporal tracking error (lag).

For each test phase, the results were averaged across the three test trials per control mode, per adaptation block and per participant. Aftereffects and transfer effects were obtained by computing the differences between pre- and post-test results for the corresponding conditions. Statistical analyses were performed using repeated measures analysis of variances (ANOVAs), and one sample/paired t -tests with Bonferroni α -adjustments to correct for multiple comparisons.

Additional analyses with regards to spatial errors and spectral power differences between target and cursor can be found in the Supplemental Materials.

## Results

**Figure 2.**
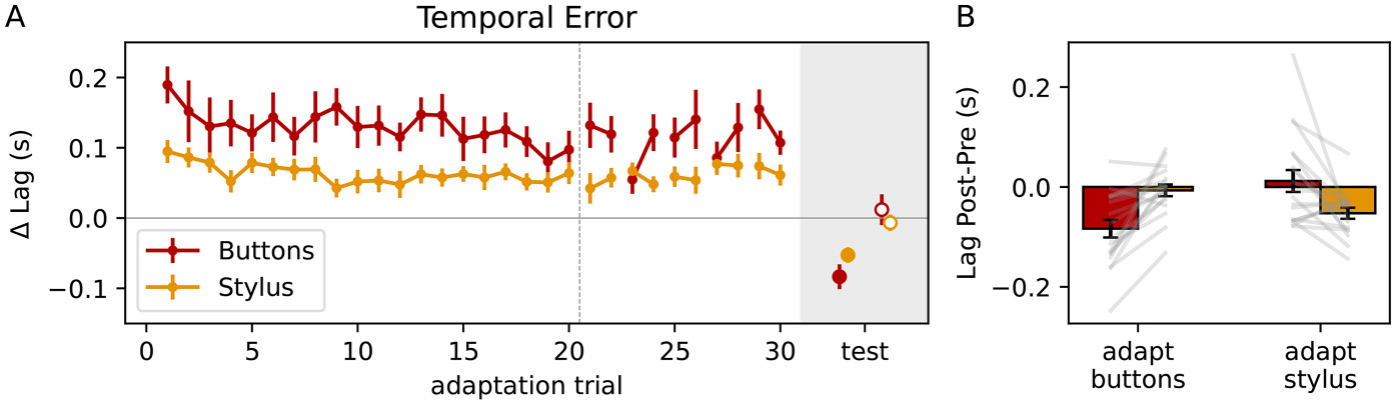
Temporal tracking lag results relative to the corresponding pre-test to take potential order effects into account (see Supplemental Material). (A) Trend over time for adaptation trials. Top-up adaptation trials are presented as a continuation of the adaptation for visualisation purposes only (after the vertical dashed line). Post-test results are shown in the grey area. Filled symbols represent the aftereffect of the control mode used during adaptation, open symbols represent transfer effects to the other control mode. (B) Post minus pre-test differences. Red and orange bars indicate Buttons and Stylus modes, respectively. Grey lines indicate individual participant results. Error bars represent standard errors of the mean in both panels.

The results of the experiment are shown in Figure 2. Figure 2A shows the trends over time during the adaptation phase for each mode in terms of the temporal tracking error (lag). In this figure the pre-test results have been subtracted from the corresponding conditions to make comparisons between Button Press and Stylus modes easier, given that they start with different baseline lags in the pre-test (Figure S2 in the Supplemental Materials). For visualisation purposes the top-up adaptation trials in the post-test are presented as a continuation of the main adaptation after the vertical dashed line (see Figure 1C for the actual sequence).

The added delay of 200 ms should initially lead to an increase in tracking lag of 200 ms, which with adaptation should decrease over time. The results indicate that the delay indeed initially leads to a detriment in performance (longer lag), which with exposure significantly improves over time (2(control mode)-by-2(trial: first vs last trial of main adaptation) RM-ANOVA; main effect trial: F(1,17)=6.97 ; p=0.017 ). Additionally, the added delay affected the Buttons Mode significantly more than the Stylus Mode (main effect control mode: F(1,17)=9.40 ; p=0.007 ). There were no significant interactions between control mode and adaptation trial.

Figure 2B shows the differences between the pre and post-test trials without the added delay. Positive values indicate a cursor that is lagging behind more than in the pre-test. Negative values indicate that the change in behaviour is towards the side of the cursor leading more than in the pre-test, which is indicative of genuine temporal recalibration in the tracking behaviour. The results clearly show a change in temporal calibration, with the cursor leading more after adaptation, but only for the control mode that was used during the adaptation phase. To test whether these effects are significant we used one-sample t -tests on the post minus pre-test results against the baseline of zero change. A post-pre difference that is significantly different from zero indicates a significant aftereffect of adaptation for the tested condition. We found a significant aftereffect for Buttons test trials (red bars in Figure 2B) after Buttons Adaptation ( t(17)=4.72 ; p=0.0002 ; α=0.0125 ), but not after adaptation using the Stylus Mode ( t(17)=0.55 ; p=0.59 ; α=0.0125 ). Similarly, the Stylus test trials (orange bars in Figure 2B) were only significantly affected by Stylus Adaptation ( t(17)=4.74 ; p=0.00019 ; α=0.0125 ) and not by Buttons Adaptation ( t(17)=0.58 ; p=0.57 ; α=0.0125 ). This indicates that temporal adaptation is specific to the control mode used during adaptation.

For completeness we also analysed the differences between the conditions shown in Figure 2B. A 2(adaptation condition: Buttons or Stylus)-by-2(test condition: Buttons or Stylus) RM-ANOVA resulted in a significant interaction between the adaptation and test modes ( F(1,17)=20.01 ; p=0.00034 ). Most particularly, there was a significant difference between the Stylus and Buttons test modes as a result of Stylus adaptation (paired-sample t -test: t(17)=2.90 ; p=0.010 ). Likewise, Buttons adaptation led to a significant difference between Button and Stylus test-modes ( t(17)=4.66 ; p=0.00022 ). This provides further support for the idea that delay adaptation is control mode specific.

## Discussion

In this experiment we investigated whether delay adaptation in a target-tracking task is specific to the mode of control of the cursor. We found that delay adaptation occurs for both controlling a cursor by moving a stylus on a graphics tablet as well as when controlling the cursor through repeatedly pressing the left and right arrow keys on a standard keyboard. However, the adapted state in terms of a shift in temporal tracking lag did not transfer to the other control mode, indicating that temporal adaptation is specific to the control mode used.

This study extends previous findings concerning the degree to which delay adaptation can transfer to different contexts. While some studies report transfer effects across tasks (Rohde et al., 2014; Sugano et al., 2012) or feedback modality (Arikan et al., 2021; Heron et al., 2009; Sugano et al., 2010, 2012), this transfer is not always complete and can be asymmetric (Arikan et al., 2021; Sugano et al., 2012). Additionally, previous studies showed that delay adaptation aftereffects appear to be stronger for active movement compared to passive movements (Kufer et al., 2024; Schmitter et al., 2023; Stetson et al., 2006). Here we show for the first time that also the mode of active sensorimotor control used during temporal adaptation matters.

These results are in line with the work by de la Malla et al. (2014) where, after adapting to a feedback delay in a target-interception task, only limited or no transfer was found for a variety of transfer tasks. It can be argued that through varying the task they may to some extent have varied the control mode. For example, in one of the transfer tasks participants had to move through a moving gap before reaching a stationary target point. This might be seen as requiring similar control as intercepting a moving target but the time-point in the movement that is most relevant for the task is different. In the interception task the end point of the movement needs to be at the right time at the right place, whereas moving through a gap would affect the planning or control midway the ballistic part of the movement. It is known that online control for ongoing movements and end-point accuracy are at least partially governed by separable processes (e.g. Sittig et al., 1985a, 1985b; van Dam & Ernst, 2013). Moreover, moving through a gap or moving across a target of the same width have been shown to be differently processed by the human sensorimotor system (Aivar et al., 2015). In fact the only condition in which de la Malla et al. (2014) found some transfer was for conditions that tested exactly the same target interception task as performed during adaptation, but for different distances between starting point and target. This could be argued to use the same control mode. This would then be in line with the study by Rohde et al. (2014) where delay adaptation transferred to a small variety of test tasks, which however all used very similar control modes and time constraints.

Mechanistically, control-mode specific delay adaptation is to some extent consistent with the idea that it occurs through a combination of adapting a movement controller and adapting the visual feedforward prediction of the expected sensory feedback (Botzer & Karniel, 2013). Temporal adjustment of the controller used, can account for the lack of transfer between control modes if assuming separate control processes for the two modes. However, our results do not necessarily provide evidence for the visual feedforward predictions being adjusted, at least not independently of control mode. If visual feedforward prediction is control-independent we should have found transfer between control modes as the task visually had exactly the same appearance in both our experimental conditions. Visual feedforward prediction can however aid in *detecting* the delay when compared to the actual visual feedback. Alternatively, delay detection could occur through comparing the visual and the proprioceptive feedback signals. Since the Buttons and Stylus control modes required different levels of involvement of wrist and fingers, the two control modes likely involved somewhat separable proprioceptive signals and thus have lead to separate estimates of cross-sensory delays between vision and proprioception. Future work will need to verify the exact mechanism for feedback delay detection and whether this leads to adaptation in the visual feedforward prediction process in a control-mode specific manner, or if it only serves to drive the temporal adjustment of the movement controller.

To conclude, though questions around the exact mechanism of delay adaptation remain, the present study is able to account for some of the differences between existing work on delay adaptation transfer, by showing that in the time domain, adaptation occurs in a control-mode specific manner.

## Supplemental Material

sj-pdf-1-ipe-10.1177_20416695251352067 - Supplemental material for Delay adaptation does not transfer between discrete button press actions and continuous control Supplemental material, sj-pdf-1-ipe-10.1177_20416695251352067 for Delay adaptation does not transfer between discrete button press actions and continuous control by Loes CJ van Dam, Svenja Kernig, Karina Lazarova, Melisa Ünal, Nicole Gappa, Benjamin Straube and Thomas SA Wallis in i-Perception

**Figure:** Video 1.

**Figure:** Video 2.
