# Supplementary material for: Delay adaptation does not transfer between discrete button press actions and continuous control
Source: Iperception. 2025 Jul 2;16(4):20416695251352067. doi: 10.1177/20416695251352067 (PMC12235500; doi:10.1177/20416695251352067)
Supplement: sj-pdf-1-ipe-10.1177_20416695251352067 - Supplemental material for Delay adaptation does not transfer between discrete button press actions and continuous control [file sj-pdf-1-ipe-10.1177_20416695251352067.pdf]

Supplemental materials to “Delay adaptation does not transfer between discrete button press actions and continuous control”

Supplemental Materials: Example trajectories

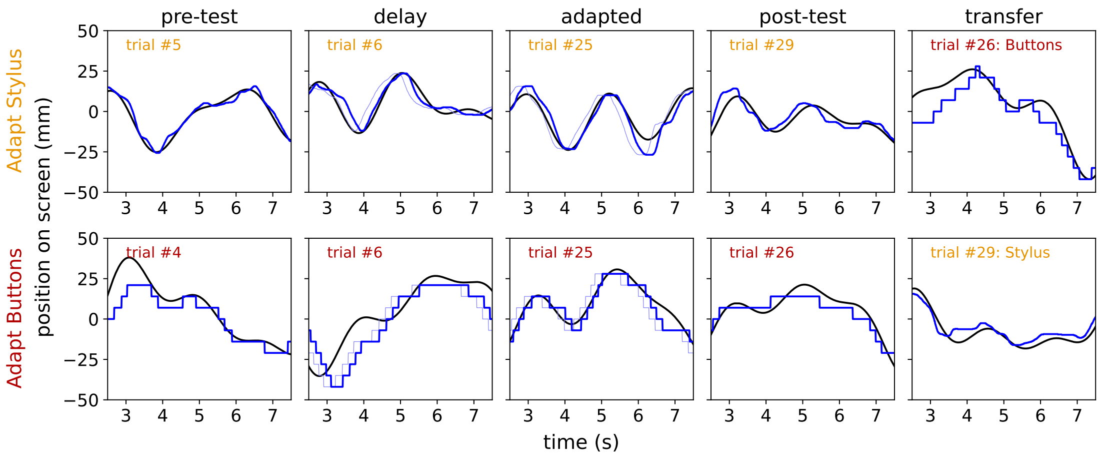

Fig S1. Example participant raw target and cursor trajectories. Each panel shows a 5 seconds excerpt from a key trial in the adaptation process for the Stylus Adaptation block (top row) and Buttons Adaptation block (bottom row). From left to right the key trials shown are: the last test trial before adaptation from the corresponding control mode; the first adaptation trial; the last adaptation trial before going into the post-test stage; the first post-test trial after adaptation using the corresponding control mode; the first post-test trial testing for transfer to the other control mode. The black line represents the target trajectory; the dark blue line the visual cursor trajectory; the light blue line (where relevant) shows where the cursor would have been without the delay.

Supplemental Materials: Pre-test analysis

Results

Before analysing the delay-adaptation aftereffects and transfer effects, we first analysed the pre-test results to determine general performance differences between the Buttons and Stylus control modes, as well as to test for potential order effects between the two testing blocks

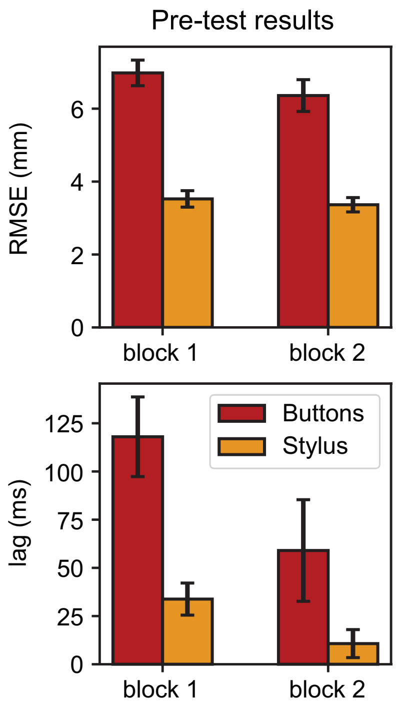

Fig S2. Pre-test results of the two blocks in the order that they were performed (regardless of the adaptation mode).

(Figure S2). Performance for Buttons trials was worse in terms of both a larger spatial Root-Mean-Squared-Error (RMSE) ($F(1, 17) = 158.49$; $p \ll 0.0001$) and lag compared to Stylus trials ($F(1, 17) = 12.38$; $p = 0.0026$). An order effect was only observed for the lag, where the lag in the second block was significantly smaller for both control modes ($F(1, 17) = 12.41$; $p = 0.0026$). We found no significant interactions between block and control mode (RMSE: $F(1, 17) = 1.09$; $p = 0.31$; lag: $F(1, 17) = 1.97$; $p = 0.18$) that could interfere with the purpose of our experiment.

For the main results, the influence of the order effects was minimised in two ways. First, as explained in the main text, the order between Stylus Adaptation and Buttons Adaptation was counterbalanced across participants such that the influence of the order effects are minimised when averaging across participants. Second, before averaging results across participants we subtracted the results of the pre-tests from the corresponding data of the same block (per control

mode). Thus, the pre-test results served as baselines only for that particular block and this way any difference in starting RMSE or lag is cancelled before averaging results across participants.

Supplemental Materials: Analysis of spatial error

To analyse potential behavioural effects and strategies when dealing with the delays, such as overshooting and cutting corners when tracking the target, we analysed the spatial errors between target and cursor as well as the power spectra of target and cursor movement (see Rohde et al. (2014)). Below we look at each of these in turn before discussing potential implications of the findings for dealing with delays.

Spatial Errors, RMSE

For the spatial errors we computed the Root-Mean-Squared-Errors (RMSE) between target and cursor. It has to be noted though that part of the spatial error will result from the temporal tracking lag. So besides a raw-RMSE we additionally computed a corrected-RMSE for which we first aligned target and cursor with respect to the observed tracking lag for a given trial, before computing the RMSE from the remaining spatial tracking errors between target and cursor. Any remaining spatial errors after correcting for the lag, are then the result of more general spatial behaviours in the tracking task such as overshooting or undershooting the target movement.

The results for the raw-RMSE and corrected-RMSE are shown in Figure S3. Figure S3A,C shows the trends over time during the adaptation phase for each mode in terms of the raw spatial tracking error (raw-RMSE, top) and the spatial error after correcting for the tracking lag (corrected-RMSE, bottom), respectively. Note that these trends are shown relatively to the corresponding result in the pretest, to make comparison between Button and Stylus trials easier, given the different baseline performance at zero delay (see Figure S2 above). The time-trend in Figure S3 shows that the delay initially leads to a detriment in performance (larger RMSE), which with exposure significantly improves over time (2(control mode)-by-2(trial: first vs last

trial of main adaptation) RM ANOVA; main effect trial: raw-RMSE $F(1, 17) = 8.91$; $p = 0.0083$; corrected-RMSE $F(1, 17) = 8.85$; $p = 0.0085$). There were no significant interactions between control mode and adaptation trial.

Figure S3B,D shows the differences between the pre and post-test trials without delay. For the spatial error the results show a trend for increased RMSE after adaptation (worse spatial performance post versus pre-test), however this was only significantly different from zero change for the Stylus mode after Stylus Adaptation (raw-RMSE: $t(17) = 5.17$; $p = 0.000077$; $\alpha = 0.0125$; corrected-RMSE: $t(17) = 5.12$; $p = 0.000086$; $\alpha = 0.0125$) and there were no significant differences or interactions between the adaptation modes and test modes. The slight increase

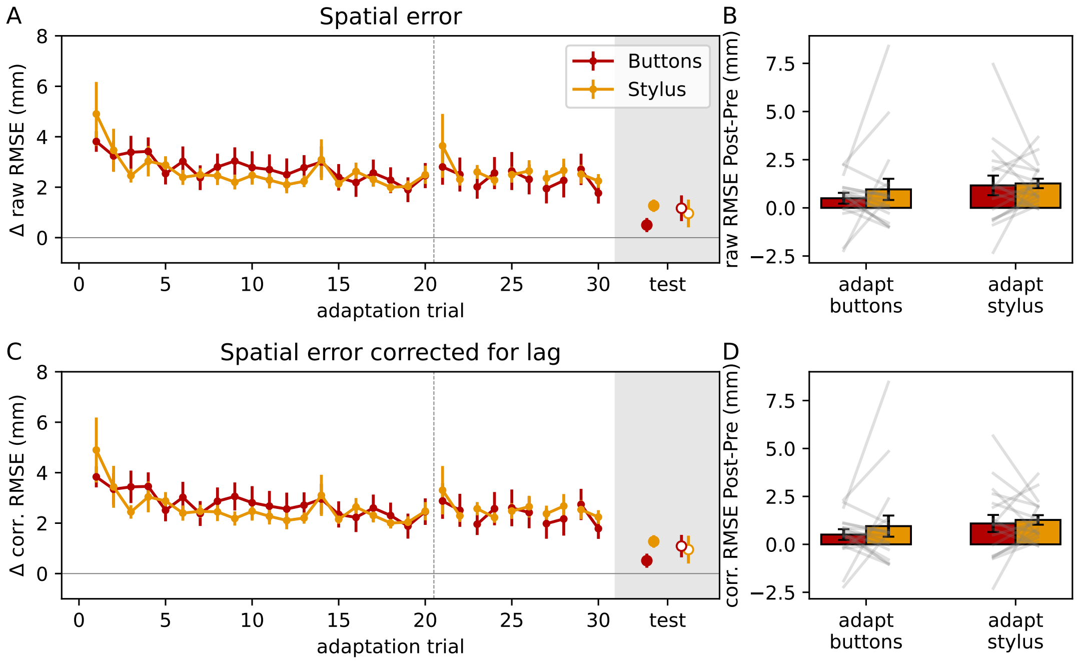

Fig S3. Spatial error between target and cursor. A,C) shows the adaptation trend over the consecutive trials for the raw-RMSE and corrected-RMSE respectively (layout the same as for Figure 2 in the main text: red lines and symbols indicating Buttons trials and orange lines and symbols indicating Stylus trials). B,D) shows the aftereffect in terms of spatial error for the raw-RMSE and corrected-RMSE respectively. Red bars indicate Buttons trials, orange bars indicate Stylus trials.

in spatial error after adaptation might be explained by participants learning to cut corners during adaptation (see also Fourier spectrum analysis below), which would affect spatial errors in particular. Though cutting corners can be a strategy to increase spatial performance, this by itself does not necessarily indicate a recalibration in the temporal sense.

In this light it is also of interest to note that the differences between the raw-RMSE (Figure S3A,B) and corrected-RMSE (Figure S3C,D) are very small. This indicates that the spatial tracking error is dominated by spatial aspects of the tracking task (e.g. overshooting the target as a result of the delay, undershooting as a compensatory strategy), rather than a direct result of the increased temporal tracking lag. The Power Spectrum analysis below was performed to gain more insights into the overshooting and undershooting behaviour in response to the target movement and/or the visuomotor delay.

Power Spectra

For the power spectra we roughly followed the analysis by Rohde et al. (2014) and mainly focused on the highest frequency component of the target movement (0.495 Hz) since this frequency is largely responsible for the noticeable turning points in the path. Therefore, the power difference between target and cursor at this frequency is indicative of systematic overshooting (more power in cursor) or undershooting (less power in cursor) the target path turning points.

Both target and cursor paths were first filtered using a Hanning window before applying the fast Fourier transform on each trajectory separately. The absolute values of the outcomes are indicative of the power at the respective frequencies. Example power spectra for the different phases in the experiment are shown in Figure S4. Note, that the frequency resolution for test and adaptation trials differs as this depends on the number of samples included which was a larger number for adaptation trials because of the longer trial duration. The frequency 0.492 Hz, which is reasonably close to the frequency of interest (0.495 Hz), is however contained in the spectrum for both types of trials. To compute the power difference between target and cursor we

took the log_{10} of the power output at the frequency of 0.492 Hz (see above), and then subtracted the resulting value for the target from the value of the cursor on a given trial (note that this is analogous to taking the ratio without the log-transform). These resulting differences are shown in Figure S5.

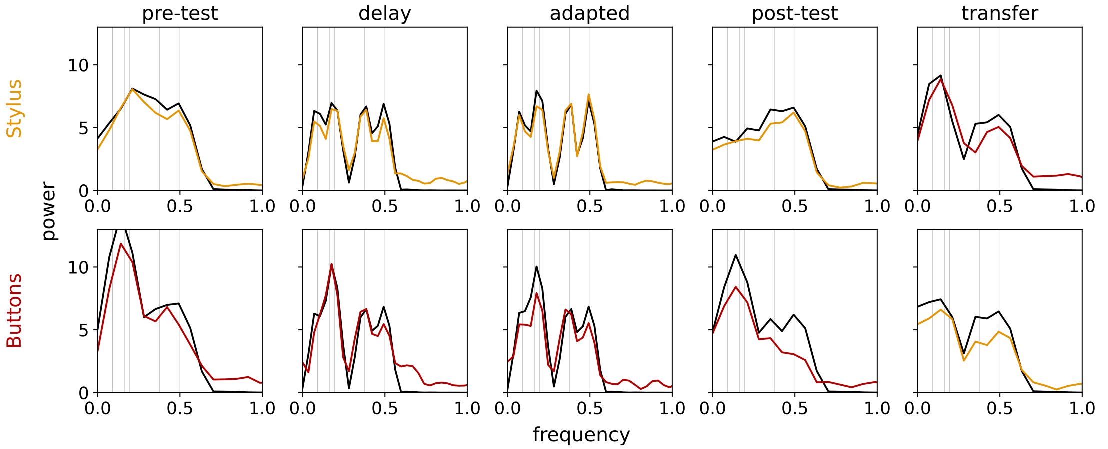

Fig S4. Example frequency spectra for target and cursor trajectories. Example normalised spectra are shown for the same participant as shown in Figure S1. Here rather than showing results for individual trials we have computed summed spectra across 3 trials for each of the panels for illustrative purposes. From left to right, we have done this for the following stages: the three pre-test trials for the control mode corresponding to the adaptation phase; the first three adaptation trials; the last three adaptation trials before the post-test; the three post-test trials for the control mode corresponding to the adaptation mode; the three test trials for the transfer control mode. The top row shows results for the Stylus Adaptation block, the bottom row for the Buttons Adaptation block. Black lines show the spectrum of the target movement, coloured lines that of the cursor (red lines indicate Buttons trials, orange line Stylus trials). The grey lines in each panel indicate the frequencies used for the generation of the target path which was a sum of non-harmonic sine waves (see Methods in main text).

Figure S5A shows how the amount of overshooting (higher power in cursor compared to target) or undershooting (lower power) changes over the course of the adaptation phase. What can be seen is that, compared to the pretest, participants tended to undershoot the target turning points a bit more for the buttons control mode when the delay was introduced. For the stylus mode participants rather overshot the target a bit more as a result of the delay. Across trials no particular trend over time can however be observed.

Figure S5B shows the aftereffect for the power at roughly 0.495 Hz (i.e. it shows the differences between pre and post tests). The negative values indicate that after adaptation, participants tended to cut corners (i.e. undershoot the target) more than before, which might in part account for the somewhat larger spatial errors after adaptation. This effect however did not significantly depend on the adaptation or test condition (Main effect adaptation mode: $F(1, 17) = 0.59$; $p = 0.45$; Main effect test mode: $F(1, 17) = 4.05$; $p = 0.06$; Interaction: $F(1, 17) = 0.016$; $p = 0.90$). To test if an individual condition reached significance we used one-sample t-test against zero-change between pre and post tests. For the Buttons test control mode this showed a trend for the power being reduced in the post-test compared to the pre-test (Buttons test trials after Buttons Adaptation: $t(17) = 2.44$; $p = 0.026$; Buttons test after Stylus Adaptation: $t = 2.75$; $p = 0.014$). However, it has to be noted that this result would not necessarily survive Bonferroni-correction using $\alpha = 0.0125$.

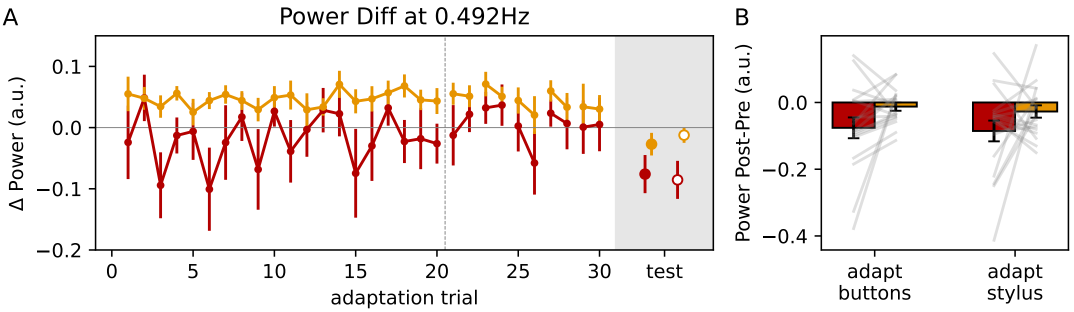

Fig S5. Power difference between target and cursor at roughly 0.495 Hz. A) shows the adaptation trend over the consecutive trials (layout the same as for Figure 2 in the main text: red lines and symbols indicating Buttons trials and orange lines and symbols indicating Stylus trials). B) shows the aftereffect in terms of power spectrum. Red bars indicate Buttons trials, orange bars indicate Stylus trials.

Insights from the spatial error analyses

Taking the results of the spatial tracking error (RMSE) and power analyses together it would seem that a spatial strategy for dealing with the delay is to cut corners at the turning points

in the target movement. This is sensible strategy, particularly for the button press mode as there will have been an upper limit for the rate at which participants can repeatedly press the buttons. This will have somewhat limited the extent to which they could follow the more extreme positions of the target, and a sensible strategy is to wait closer to the centre of the display for the target to return. This also explains why the button-press mode generally leads to worse spatial tracking performance even in the pretest when compared to the stylus where the tracking is more continuous (Figure S2). With the added delay, increasing the use of cutting corners (as can be observed in Figure S5) can help to keep spatial performance in an acceptable range, despite the delay. This strategy then leads to some improvement in spatial tracking (see Figure S3), irrespective of the additional adjustments in the temporal tracking lag as shown in the main text (Figure 2 main text).

References

Rohde M, van Dam L and Ernst M (2014) Predictability is necessary for closed-loop visual feedback delay adaptation. J. Vis 14: 1–23. DOI:10.1167/14.3.4.
